# Supplementary material for: Alternative strategies for closing the award gap between white and minority ethnic students
Source: eLife. 2021 Aug 4;10:e58971. doi: 10.7554/eLife.58971 (PMC8337073; doi:10.7554/eLife.58971)
Supplement: Transparent reporting form [file elife-58971-transrepform.docx]

***eLife’s* transparent reporting form**

We encourage authors to provide detailed information *within their submission* to facilitate the interpretation and replication of experiments. Authors can upload supporting documentation to indicate the use of appropriate reporting guidelines for health-related research (see [EQUATOR Network](http://www.equator-network.org/%20)), life science research (see the [BioSharing Information Resource](https://biosharing.org/)), or the [ARRIVE guidelines](http://www.plosbiology.org/article/info:doi/10.1371/journal.pbio.1000412) for reporting work involving animal research. Where applicable, authors should refer to any relevant reporting standards documents in this form.

If you have any questions, please consult our Journal Policies and/or contact us: [editorial@elifesciences.org](mailto:editorial@elifesciences.org).

**Sample-size estimation**

- You should state whether an appropriate sample size was computed when the study was being designed
- You should state the statistical method of sample size computation and any required assumptions
- If no explicit power analysis was used, you should describe how you decided what sample (replicate) size (number) to use

Please outline where this information can be found within the submission (e.g., sections or figure legends), or explain why this information doesn’t apply to your submission:

Sample-size estimation does not apply to the submission as the study looked at entire populations. However, as described in the results, year 3 courses are inherently smaller than year 1 and 2 classes. Therefore, the relatively larger year 3 courses formed part of the basis for their selection. This information is located in *Results – Exploration…section*.

**Replicates**

- You should report how often each experiment was performed
- You should include a definition of biological versus technical replication
- The data obtained should be provided and sufficient information should be provided to indicate the number of independent biological and/or technical replicates
- If you encountered any outliers, you should describe how these were handled
- Criteria for exclusion/inclusion of data should be clearly stated
- High-throughput sequence data should be uploaded before submission, with a private link for reviewers provided (these are available from both GEO and ArrayExpress)

Please outline where this information can be found within the submission (e.g., sections or figure legends), or explain why this information doesn’t apply to your submission:

The data is not biological, however comparable considerations on replicates apply. The equivalent of replica is number of individual sets of data (12 total - eight in the year 1-2 pathway and four in the year 3 study). Details of these sets of data are described in the *Exploration…* section of the results.

One outlier was identified (*Figure 2C*, year 2, cohort 4) and discussed. A test of why the outlier existed was performed and the results reported in the *Results – Dissecting Cell Biology Components…* text.

All criteria for inclusion, exclusion were carefully described in the *Exploration…* section of the results and *Students* *Included…* section of the methods. Number excluded is further stated in the figure legends.

Further in-depth evaluation of the study design and characteristics was made and reported in the *Results – Exploration…section*. These demonstrate rigorous procedures.

**Statistical reporting**

- Statistical analysis methods should be described and justified
- Raw data should be presented in figures whenever informative to do so (typically when N per group is less than 10)
- For each experiment, you should identify the statistical tests used, exact values of N, definitions of center, methods of multiple test correction, and dispersion and precision measures (e.g., mean, median, SD, SEM, confidence intervals; and, for the major substantive results, a measure of effect size (e.g., Pearson's r, Cohen's d)
- Report exact p-values wherever possible alongside the summary statistics and 95% confidence intervals. These should be reported for all key questions and not only when the p-value is less than 0.05.

Please outline where this information can be found within the submission (e.g., sections or figure legends), or explain why this information doesn’t apply to your submission:

Data in *Figure 1* is the mean of 3-individual cohorts. The errors bars in the graphs are plus, minus standard error of the mean and this is defined in the *Figure 1 - Legend*. All numerical values are provided in *Figure 1 – Source data 1*.

The major results are expressed as the original raw data (percentage of students awarded good grades) for each individual cohort or course (*Figure 2A-D*). (Obviously) there are no error bars with this type of absolute data. There are a large number of exact N values and these are given in *Figure 2 – source data 1* or in the body of the submission. Mean proportion and SEM of BAME and White students that are awarded good grades are provided in *Figure 2 – Source data 1.*

Average award gap (*Figure 2E, F*) is the difference between these mean proportions. This definition is provided in the *Results – Exploration*…section, *Figure 2 – Source data 1 and methods*. Exact values of Cohen’s d are located in *Figure 2- Source data 1* and the data summarized in the main *Results* tex*t*.

Data for the entire university are either provided in the *Results* text, or *Figure 2F* as above. Numerical values for *Figure 2F* and related means and SEM are provided in *Figure 2- Source dat*a 1.

Other main substantive results, are frequencies of each mark (from <30% to 100% at 5% intervals) awarded to students (*Figure 3*). This is also raw data and is the frequency aggregate across specified cohorts (stated in *Figure 3 legend*, results and methods; and the cohorts included are listed in *Figure 3 – source data 1 and Figure 3 - legend*). One cohort (the outlier, see replicate section, above) was excluded and explained in *Figure 3,* figure legend. For fair analysis (so as to be able to directly compare pattern of distribution of marks where BAME and White cohort size varies), raw frequency for BAME students was normalised to White student cohort size (the larger cohort). Normalization factors and exact student raw count (N) for each cohort and each component are shown in *Figure 3 – figure supplement 1- source data*. This makes transparent the raw data used for the calculation of each normalisation factor.

Confidence interval is a measure of how near the sample mean (e.g. height of 10 year-old girls, in a sample of 20) is to the population mean (if one could measure the height of all 10 year old girls). However, the data in this paper is not a sample, but *is* the finite population. Standard error of the mean is therefore the most appropriate measure of accuracy of the mean (and provided, as above). Pearson’s r does not apply because this measures how closely correlated two sets of variables are to each other; but there is no such analysis in the submission (and none that exists in the context of the study).

Statistical tests of significance were not applied because of the relatively low N number, as reported in the *Methods* and following guidance provided in two reports in Nature and Nature methods (cited in the *Methods*).

(For large datasets, or papers with a very large number of statistical tests, you may upload a single table file with tests, Ns, etc., with reference to sections in the manuscript.)

**Group allocation**

- Indicate how samples were allocated into experimental groups (in the case of clinical studies, please specify allocation to treatment method); if randomization was used, please also state if restricted randomization was applied
- Indicate if masking was used during group allocation, data collection and/or data analysis

Please outline where this information can be found within the submission (e.g., sections or figure legends), or explain why this information doesn’t apply to your submission:

Group allocation does not apply. When a student enrolls at UCL they provide their ethnicity and that process is independent of this study. When data is subsequently extracted for any given study (like this one), student characteristics as defined in the university’s records come with the extraction. This is explained in the methods.

**Additional data files (“source data”)**

- We encourage you to upload relevant additional data files, such as numerical data that are represented as a graph in a figure, or as a summary table
- Where provided, these should be in the most useful format, and they can be uploaded as “Source data” files linked to a main figure or table
- Include model definition files including the full list of parameters used
- Include code used for data analysis (e.g., R, MatLab)
- Avoid stating that data files are “available upon request”

Please indicate the figures or tables for which source data files have been provided:

Figure 1 – Source data 1, including exact N

Figure 2 – Source data 1, including exact N

Figure 3 – Exact N and normalization frequencies are located in Figure 3 – Figure supplement 1 – Source data 1

Figure 3 – Figure supplement 1 – Source data 1, including exact N

Figure 4 – Source data 1, including exact N

Data on evidence informing recommendations – main text and *Methods*
